# Sebaceous Adenoma of the Caruncle Mimicking Papilloma: Clinicopathological Features and the Ophthalmic Relevance of Mismatch Repair Immunohistochemistry

**DOI:** 10.7759/cureus.113170

**Published:** 2026-07-22

**Authors:** Imogen Cheung, David Cheung

**Affiliations:** 1 Ophthalmology, University of Leicester, Leicester, GBR; 2 Ophthalmology, Birmingham and Midland Eye Centre, Birmingham, GBR

**Keywords:** caruncle, immunohistochemistry, lynch syndrome, mismatch repair, muir-torre syndrome, ocular adnexal tumours, sebaceous adenoma

## Abstract

Sebaceous adenoma is a rare benign sebaceous neoplasm that only exceptionally involves the ocular adnexa. Caruncular lesions are particularly uncommon and often resemble more frequently encountered lesions such as papilloma. Correct diagnosis of these tumours is important because sebaceous neoplasms may be associated with Muir-Torre syndrome (MTS), a hereditary cancer predisposition syndrome within the Lynch syndrome spectrum. Modern pathology reports incorporate mismatch repair (MMR) immunohistochemistry to help identify patients who may require genetic assessment and cancer surveillance. We present the case of an 81-year-old woman who presented with a right caruncular lesion associated with epiphora and mucoid discharge. Clinical examination demonstrated a cream coloured papillomatous lesion that was mobile and not adherent to adjacent structures. The lesion was presumed clinically to represent a benign papilloma and was excised during combined lacrimal surgery. Histopathological examination demonstrated a well-circumscribed lobulated sebaceous proliferation composed predominantly of mature sebocytes with peripheral basaloid germinative cells and no significant atypia, consistent with sebaceous adenoma. Immunohistochemistry demonstrated preserved nuclear expression of MutL Homologue 1 (MLH1), postmeiotic segregation increased 2 (PMS2), MutS homologue 2 (MSH2) and MutS homologue 6 (MSH6), indicating intact MMR function and reducing suspicion for MTS. Sebaceous adenoma should be considered in the differential diagnosis of cream-white papillomatous caruncular lesions. Ophthalmologists should understand the significance of MMR immunohistochemistry, as abnormal results may identify patients requiring genetic referral, whereas preserved expression is generally reassuring and supports a sporadic lesion. Retained nuclear staining for MLH1, PMS2, MSH2 and MSH6 indicated preserved MMR function (MMR proficient; pMMR), which predicts microsatellite stability (MSS). In conjunction with the patient’s age and absence of a personal history of malignancy, these findings strongly favoured a sporadic sebaceous adenoma. Although intact MMR expression does not completely exclude MTS, abnormal staining would have prompted consideration of genetic referral and systemic evaluation. Our report expands upon previous literature by providing a practical explanation of MMR immunohistochemistry aimed at ophthalmologists, who may be the first clinicians to recognise sebaceous neoplasia and initiate appropriate MTS screening.

## Introduction

The caruncle is a unique structure situated within the medial canthus of the eye. Unlike most of the conjunctiva, it contains both conjunctival and cutaneous elements, including hair follicles, sebaceous glands and accessory lacrimal tissue. Consequently, a broad range of lesions may arise from this small anatomical area [1].

Sebaceous adenoma is a benign epithelial tumour exhibiting sebaceous differentiation. Although relatively well recognised in dermatopathology, ocular involvement is distinctly uncommon. Sebaceous adenoma accounts for approximately 1% of caruncular lesions [2] and is encountered much less frequently than papillomas, naevi and oncocytomas. Most published ophthalmic reports consist of isolated case reports and small case series [3].

The importance of recognising sebaceous adenoma extends beyond confirming a benign diagnosis. Sebaceous neoplasms may occasionally represent the first manifestation of Muir-Torre syndrome (MTS), an inherited cancer predisposition syndrome characterised by sebaceous tumours and internal malignancies [4]. We report an unusual case of caruncular sebaceous adenoma initially presumed clinically to represent a papilloma. To our knowledge, this is among the few reported cases of caruncular sebaceous adenoma assessed with mismatch repair (MMR) immunohistochemistry and one of the few ophthalmic reports to provide a practical explanation of MMR interpretation directed at ophthalmologists.

## Case presentation

An 81-year-old woman was referred to the oculoplastic service with a lesion involving the right medial canthus. She complained of chronic mucoid discharge arising from the lesion together with mild epiphora.

**Figure 1:**
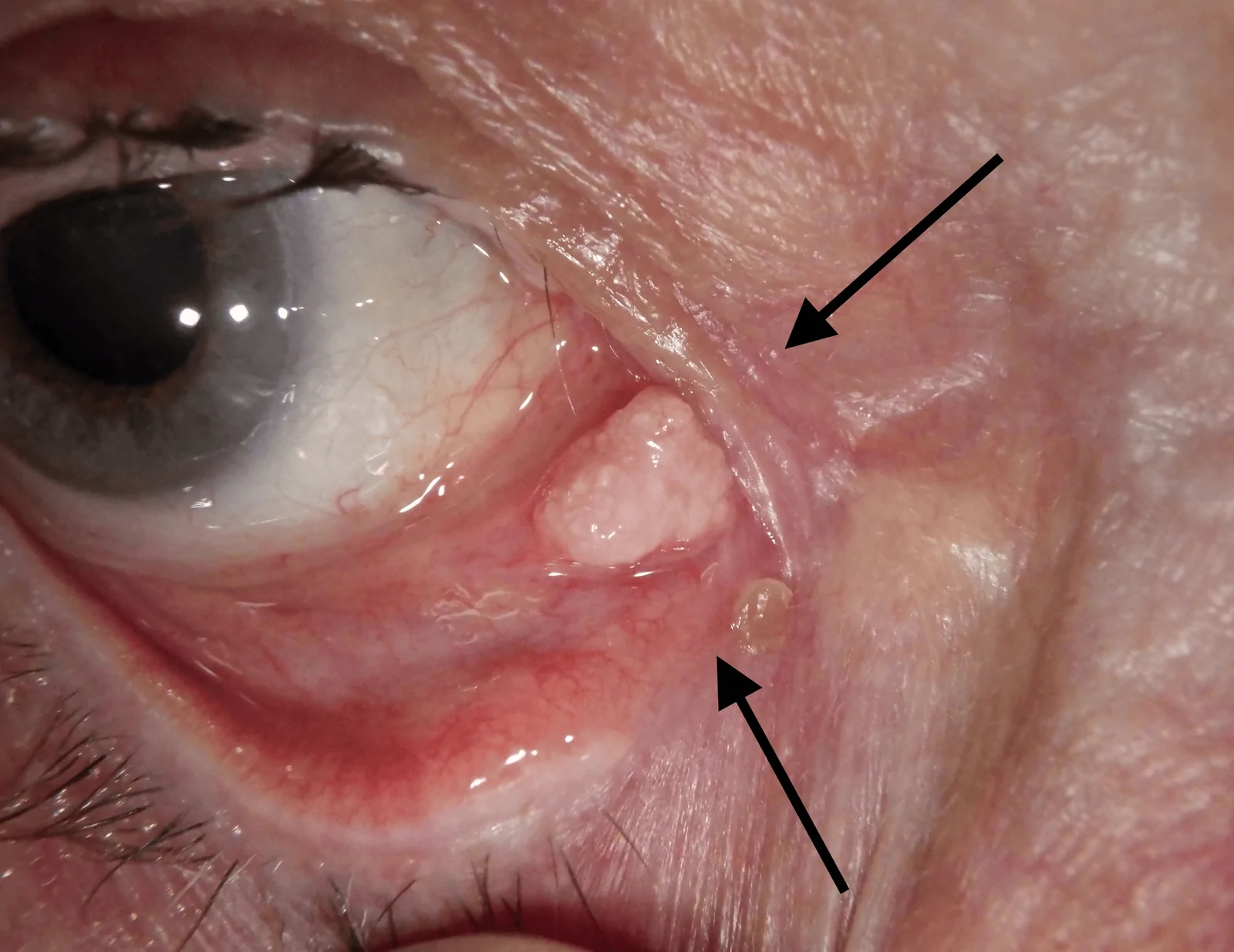
Clinical photograph demonstrating a pale yellow-white, lobulated papillomatous lesion arising from the right caruncle, with coincidental punctal stenosis (arrows).

Best-corrected visual acuity measured 6/7.5 in both eyes. Intraocular pressures were normal. Examination demonstrated a pale yellow-white lobulated papillomatous lesion arising from the right caruncle (Figure 1). The lesion was mobile and not adherent to any surrounding structures. She was also coincidentally found to have punctal stenosis and punctal ectropion. The remainder of the ocular examination was unremarkable.

Clinically, the lesion was presumed to represent a benign papilloma. However, given the uncertainty of the diagnosis and the patient’s symptoms, excisional biopsy was recommended, combined with simultaneous lacrimal surgery to address the epiphora.

Following informed consent, the patient underwent right carunculectomy together with right lower lid syringing and probing and three-snip punctoplasty under local anaesthesia. At three-week follow-up, the caruncular wound had healed well, and the watering had resolved. The patient had an uneventful recovery with no evidence of recurrence of the lesion after two years of follow-up.

Histology

**Figure 2:**
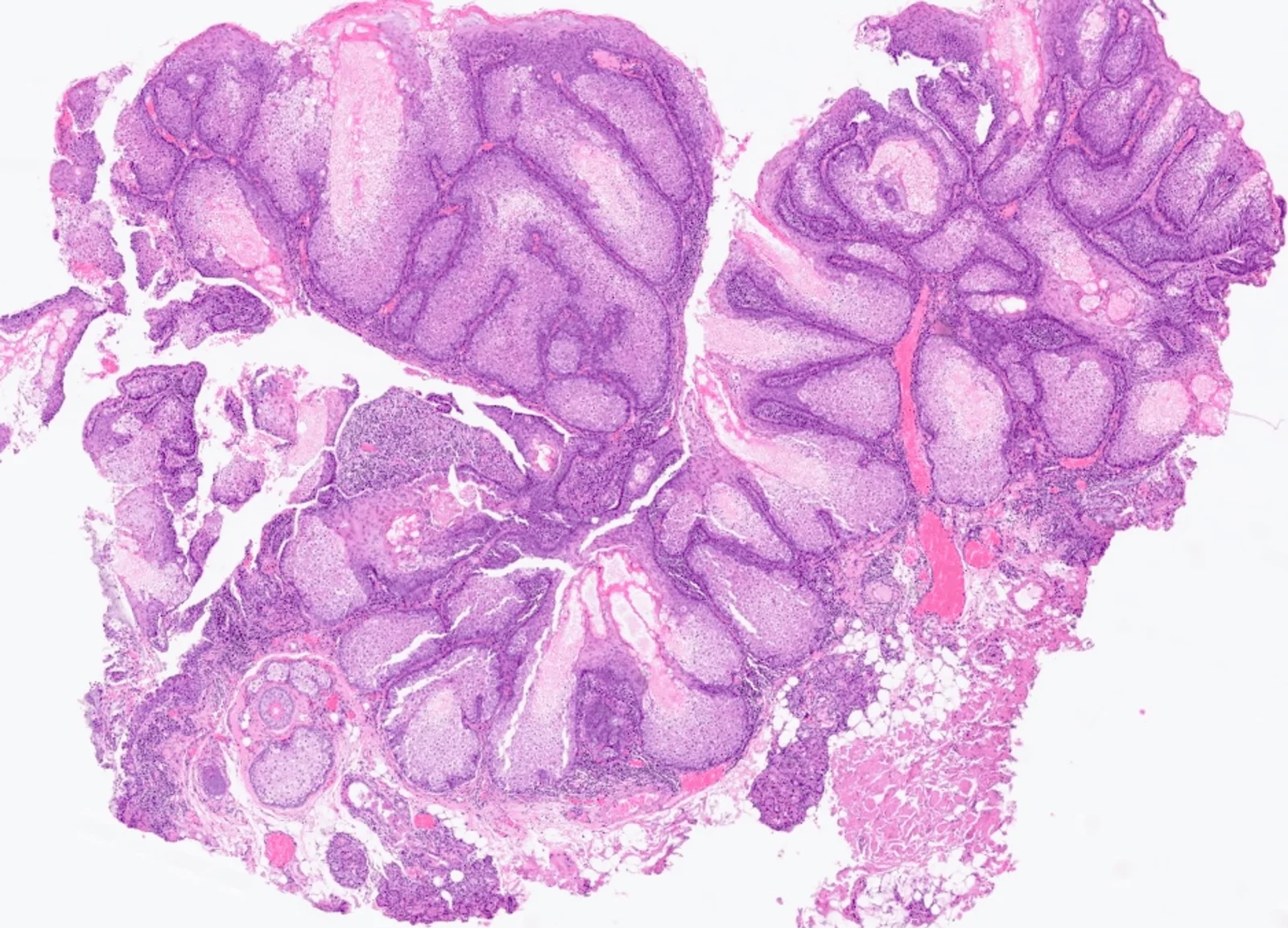
Low-power (80x magnification) haematoxylin and eosin photomicrograph demonstrating a well-circumscribed, lobulated sebaceous proliferation.

**Figure 3:**
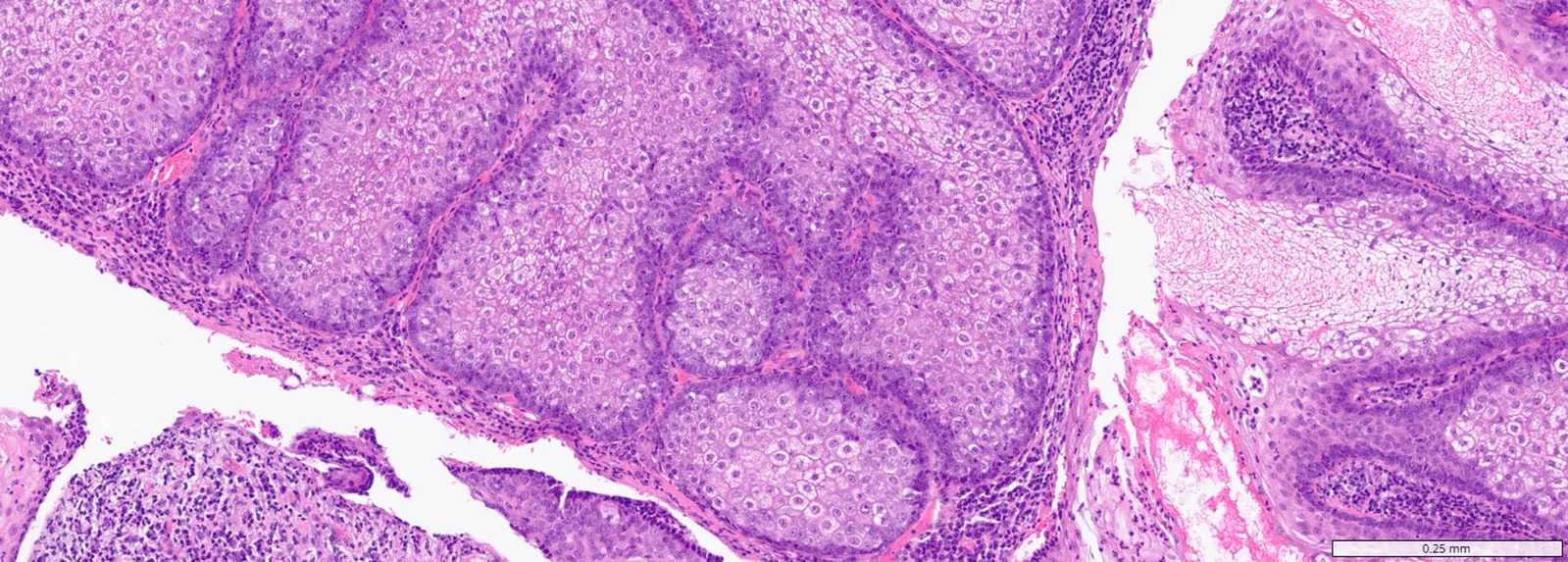
Higher-power (400x magnification) haematoxylin and eosin photomicrograph showing mature sebocytes with abundant vacuolated cytoplasm and peripheral rims of small basaloid germinative cells without significant atypia. The vacuolated appearance resulted from dissolution of intracellular lipid during routine tissue processing.

Macroscopic examination demonstrated a warty tissue fragment measuring 5 × 5 × 5 mm. Histopathological examination revealed a well-circumscribed, completely excised lobulated sebaceous proliferation beneath the conjunctival epithelium (Figures 2-3) with clear margins. Numerous enlarged sebaceous lobules were separated by delicate fibrous septa.

**Figure 4:**
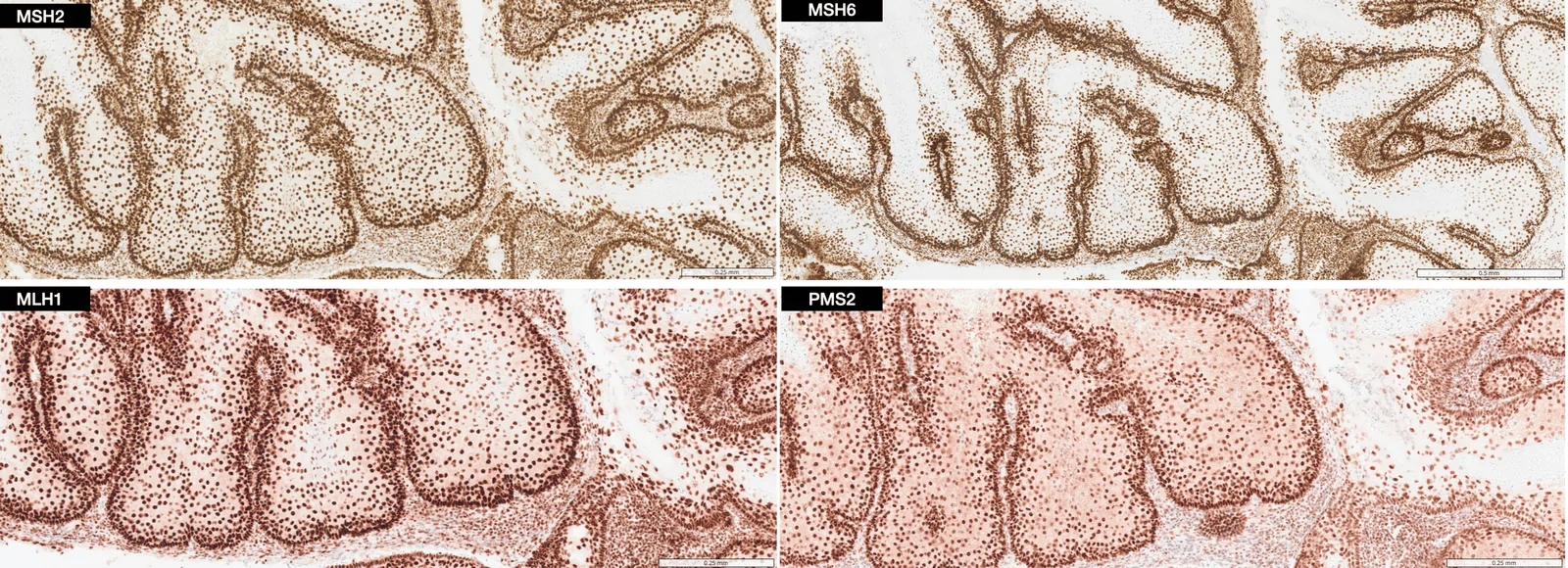
Montage of immunohistochemical staining demonstrating preserved nuclear expression of MLH1, PMS2, MSH2 and MSH6 proteins in the sebocytes (400x magnification). Internal positive controls were present.

Importantly, mature sebocytes predominated over basaloid elements. There was no significant cytological atypia, increased mitotic activity, necrosis, infiltrative growth or pagetoid spread (differentiating it from sebaceous carcinoma). These features were diagnostic of sebaceous adenoma. The sample was also subjected to immunohistochemical analysis (Figure 4).

## Discussion

Every time a cell divides, it must copy approximately three billion DNA base pairs. Unsurprisingly, occasional “spelling mistakes” occur during this copying process. Fortunately, cells possess an internal proofreading system known as the MMR pathway, whose role is to identify and correct these errors before they become permanent mutations [5]. Four proteins play an important role in this process: MutS homologue 2 (MSH2), MutS homologue 6 (MSH6), MutL Homologue 1 (MLH1) and postmeiotic segregation increased 2 (PMS2). MSH2 and MSH6 work together as cellular “spelling-checkers” patrolling newly copied DNA. Once an error has been recognised, MLH1 and PMS2 coordinate the repair process by removing the faulty DNA segment and replacing it with the correct sequence. Together, these proteins function as the cell’s quality-control system, maintaining genomic stability [6]. If any of these proteins are absent because of pathogenic variants affecting the corresponding MMR genes, DNA replication errors will slowly accumulate. These errors occur particularly within short repetitive DNA sequences known as microsatellites, leading to insertions and deletions giving rise to a characteristic molecular signature termed microsatellite instability (MSI) [7]. Deficient MMR and the resulting MSI phenotype underpin Lynch syndrome, an autosomal dominant syndrome associated with an increased risk of gastrointestinal, endometrial, ovarian and genitourinary malignancies. When Lynch syndrome occurs in association with sebaceous neoplasms, it is referred to as MTS [8]. Loss of any of the four MMR proteins may occur, although pathogenic variants in MSH2 predominate in MTS and typically result in loss of MSH2 and MSH6 staining [9].

Our patient’s tumour showed retained nuclear expression of all four MMR proteins and was therefore considered MMR-proficient (pMMR). Although pMMR is generally associated with microsatellite stability, MSI testing was not performed in this case. A recent meta-analysis reported a sensitivity of approximately 84% and specificity of only 46% for MMR immunohistochemistry in detecting MTS; importantly, most MMR-deficient sebaceous neoplasms were not attributable to MTS [10]. Retained staining therefore reduces, but does not eliminate, the possibility of an underlying germline MMR variant, while loss of staining is not itself diagnostic of MTS. The result must be interpreted alongside the patient’s personal and family history and, where appropriate, the Mayo MTS risk score [11]. The Mayo MTS risk score incorporates age at presentation, the number of sebaceous neoplasms at presentation and any personal or family history of Lynch-associated malignancy [11]. Our patient was 81 years old, had a single sebaceous neoplasm and had no personal or family history of a Lynch-associated malignancy, giving a Mayo MTS risk score of 0. Loss of one or more proteins would have prompted genetic referral and systemic evaluation [12], but equally, referral may still be appropriate despite intact staining if the clinical history remains strongly suggestive [13,14]. In some patients, the sebaceous lesion precedes the diagnosis of visceral malignancy by several years [15]. It is therefore vital that surgeons dealing with these tumours recognise their importance in ongoing patient referral and management.

Sebaceoma also enters the histological differential diagnosis because both lesions contain basaloid germinative cells and mature sebocytes [15]. In sebaceous adenoma, mature sebocytes predominate within well-circumscribed lobules with only a peripheral rim of basaloid cells, whereas sebaceoma is predominantly composed of basaloid germinative cells, conventionally comprising more than half of the tumour. In a recent series of benign and atypical ocular sebaceous neoplasms, Jia et al. found MMR deficiency in four of six sebaceous adenomas, whereas all five sebaceomas retained MMR protein expression [16]. In contrast to the majority of adenomas in that series, the adenoma in our patient was pMMR. These findings demonstrate that MMR status cannot be reliably inferred from histological classification alone and support individual assessment of ocular sebaceous neoplasms. In contrast to our case, most adenomas arose from the eyelid margin or tarsal conjunctiva rather than the caruncle, highlighting the rarity of our patient's presentation.

Our case shares several features with the three ocular sebaceous adenomas described by Dias et al. [3]. Their patients, aged 58-73 years, had small pink or yellow raised lesions involving the plica semilunaris or caruncle, and one presented with a watery eye. All three tumours showed the same basic histological pattern of mature sebocytes surrounded by basaloid germinative cells. MMR expression was preserved in the two cases for which staining was reported, and no recurrence or systemic malignancy was found during the available six- to 12-month follow-up in two patients. Our patient presented similarly with discharge and a raised lesion at the caruncle with similar histology and pMMR immunohistochemistry profile. She remained recurrence-free after two years. We hope the longer follow-up in our case, together with this explanation of how to interpret intact MMR staining, will add a practical ophthalmic perspective to the earlier series.

## Conclusions

Sebaceous adenoma of the caruncle is exceptionally rare and may closely mimic more common lesions encountered in ophthalmic practice. Previous case reports have been uncommon, and although they discuss the clinical features and histology, they have rarely discussed the underlying genetic significance of these lesions. Sebaceous adenoma should be considered in the differential diagnosis of pale cream-white caruncular lesions. Clinical appearance alone is insufficient for diagnosis, and excisional biopsy remains essential to help distinguish from sebaceoma and sebaceous carcinoma. Unlike sebaceous adenoma, sebaceous carcinoma exhibits infiltrative growth, marked cytological atypia, increased mitotic activity and may demonstrate pagetoid spread.

Ophthalmologists should appreciate the wider implications of diagnosing sebaceous tumours. Although most prove sporadic, sebaceous neoplasia can be the first manifestation of MTS. MMR immunohistochemistry provides a practical way of assessing this possibility, but it must be interpreted in the context of the patient's personal and family history. Retention of all four proteins is reassuring, but it does not exclude an inherited MMR abnormality completely.
